# Myocardial hypoperfusion due to hypoplastic coronary arteries: a cause for false-positive results during adenosine stress Cardiac Magnetic Resonance Imaging

**DOI:** 10.1186/1532-429X-11-S1-P155

**Published:** 2009-01-28

**Authors:** Guenter Pilz, Maximilian Graw, Tobias Heer, Markus Klos, Eman Ali, Berthold Hoefling

**Affiliations:** Cardiac MRI, Clinic Agatharied, University of Munich, Hausham, Germany

**Keywords:** Coronary Angiography, Cardiac Magnetic Resonance, Coronary Stenosis, Significant Coronary Artery Disease, Stress Cardiac Magnetic Resonance

## Background

Magnetic resonance myocardial perfusion imaging (MRMPI) based on adenosine stress cardiac magnetic resonance (CMR) is increasingly proposed for non-invasive detection of relevant coronary artery disease (CAD). However, little is known about the impact of the normal variability of coronary anatomy and caliber on the diagnostic performance of adenosine stress CMR.

## Purpose

Aim of our study was to examine whether hypoplastic coronary arteries found as normal variations in right-dominant or left-dominant circulation may account for visualization of hypoperfusion in the absence of coronary stenosis and thus may result in false-positive (FP) results of MRMPI in the assessment of CAD.

## Methods

From 05/2007 until 01/2008, we enrolled 206 consecutive patients with suspected CAD undergoing MRMPI, who were diagnosed as having myocardial ischemia during adenosine-stress perfusion and subsequently underwent coronary angiography (CA). Patients were examined in a 1.5 T whole-body scanner (GE Signa Excite). After three minutes of adenosine infusion (140 μg/kg/min), myocardial first-pass sequence in 4–5 continuous short-axis orientation using Gadolinium-based contrast agent was performed (0.1 mmol/kg, Omniscan, GE Healthcare Buchler). Images were visually analyzed in comparison to rest perfusion by two experienced investigators in consensus. A perfusion deficit was regarded relevant if affecting more than 1/3 of myocardial wall thickness in at least two neighbouring myocardial segments and persisting for more than five heart beats after maximal signal intensity in the LV cavity. Significant CAD was defined as luminal narrowing of ≥70% in CA.

## Results

**Figure 1:**
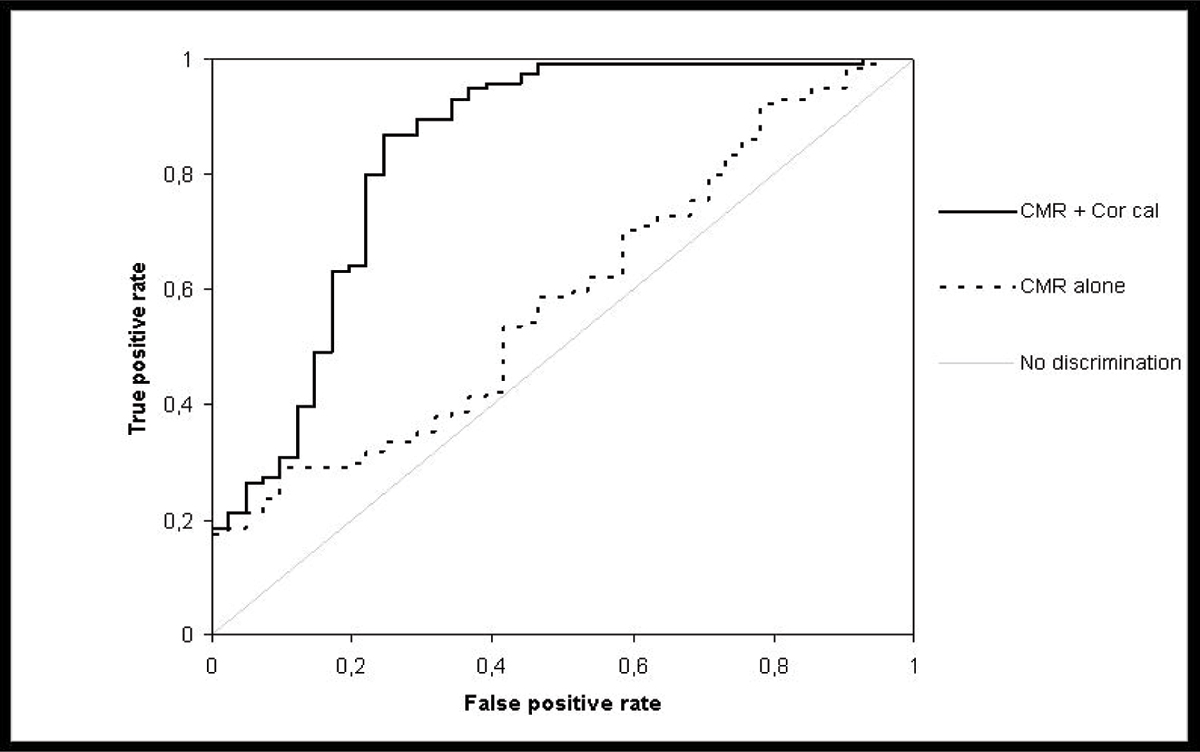
Figure 1

Significant CAD was invasively confirmed in 165 out of 206 (CMR specificity: 80.1% = true positive (TP)) and ruled out in 41 / 206 patients with previous ischemia in MPMRI (= FP). TP and FP were comparable for pre-test risk (Morise scores 15.0 vs.15.5, p = 0.32) and CMR findings (Table 1). CA confirmed that the proximal diameters of the coronary vessels correlate well with the type of coronary dominance. As the main result we found a significant correlation between FP CMR results and presence of a hypoplastic coronary vessel in CA (mean proximal diameter 2.5 ± 0.3 mm) supplying the area of ischemia in CMR, opposite to the dominant vessel. Logistic regression analysis showed the presence of a hypoplastic vessel supplying the region of ischemia to be the most predictive parameter for discrimination of FP versus TP. By adding this information to CMR, diagnostic accuracy to avoid FP improved significantly (ROC analysis). Figure 1 compares ROC curves for identification of FP patients using standard CMR parameters (see table) alone (dotted line, AUC: 0.59 ± 0.05) and the combination of standard CMR parameters and coronary caliber (Cor cal) information (presence of a hypoplastic vessel in the area of ischemia (full line, AUC: 0.84 ± 0.04, p < 0.0001)).

**Table 1:** Table 1

CMR parameter	FP results	TP results	P value
LV EF	65.8 %	65.6 %	0.86
LV EDV (ml)	126	129	0.58
LV mass (g)	130	137	0.20
LV wall stress	35.7	34.7	0.62
Transmural extent of ischemia	41.1 %	41.3 %	0.64
Temporal persistence of ischemia (beats)	8.9	8.7	0.44
CMR – CA correlation:			
Ischemia in area with hypoplastic vessel	51.2 %	0.6 %	<0.0001

## Conclusion

Hypoplastic coronary arteries found as normal variations in right-dominant or left-dominant circulation may account for visualization of hypoperfusion in the absence of coronary stenosis and thus may result in false-positive results of MRMPI in the assessment of CAD. Thus, noninvasive asessment of the proximal coronary diameter in the vessel supplying the area of ischemia could reduce the rate of false positive CMR results and the rate of subsequent superfluous diagnostic CA.

